# Strong peak immunogenicity but rapid antibody waning following third vaccine dose in older residents of care homes

**DOI:** 10.1038/s43587-022-00328-3

**Published:** 2023-01-20

**Authors:** Gokhan Tut, Tara Lancaster, Maria Krutikov, Panagiota Sylla, David Bone, Eliska Spalkova, Christopher Bentley, Umayr Amin, Azar Jadir, Samuel Hulme, Nayandeep Kaur, Elif Tut, Rachel Bruton, Mary Y. Wu, Ruth Harvey, Edward J. Carr, Crick COVID Immunity Pipeline, Bobbi Clayton, Sina Namjou, Vanessa Silva, Meghan Poulten, Philip Bawumia, Murad Miah, Samuel Sade, Mauro Miranda, Tom Taylor, Ilenia D’Angelo, Mercedes Cabrera Jarana, Mahbubur Rahman, Janet Abreu, Sandeep Sandhar, Neil Bailey, Simon Caidan, Marie Caulfield, Mary Wu, Ruth Harvey, Lorin Adams, Caitlin Kavanagh, Scott Warchal, Chelsea Sawyer, Mike Gavrielides, Jag Kandasamy, Karen Ambrose, Amy Strange, Titilayo Abiola, Nicola O’Reilly, Philip Hobson, Ana Agau-Doce, Emma Russell, Andrew Riddell, Svend Kjaer, Annabel Borg, Chloë Roustan, Christophe Queval, Rachel Ulferts, Charles Swanton, Sonia Gandhi, Steve Gamblin, Rupert Beale, Oliver Stirrup, Madhumita Shrotri, Borscha Azmi, Christopher Fuller, Verity Baynton, Aidan Irwin-Singer, Andrew Hayward, Andrew Copas, Laura Shallcross, Paul Moss

**Affiliations:** 1 Institute of Immunology and Immunotherapy, University of Birmingham, Birmingham, UK; 2 UCL Institute of Health Informatics, London, UK; 3 Covid Surveillance Unit, The Francis Crick Institute, London, UK; 4 Worldwide Influenza Centre, The Francis Crick Institute London, London, UK; 5 The Francis Crick Institute, London, UK; 6 Genotype-to-Phenotype UK National Virology Consortium (G2P-UK), London, UK; 7 UCL Department of Renal Medicine, Royal Free Hospital, London, UK; 8 Health Security Agency, London, UK; 9 Health Data Research UK, London, UK; 10 UCL Institute for Global Health, London, UK

**Keywords:** Infection, SARS-CoV-2, Ageing

## Abstract

Third-dose coronavirus disease 2019 vaccines are being deployed widely but their efficacy has not been assessed adequately in vulnerable older people who exhibit suboptimal responses after primary vaccination series. This observational study, which was carried out by the VIVALDI study based in England, looked at spike-specific immune responses in 341 staff and residents in long-term care facilities who received an mRNA vaccine following dual primary series vaccination with BNT162b2 or ChAdOx1. Third-dose vaccination strongly increased antibody responses with preferential relative enhancement in older people and was required to elicit neutralization of Omicron. Cellular immune responses were also enhanced with strong cross-reactive recognition of Omicron. However, antibody titers fell 21–78% within 100 d after vaccine and 27% of participants developed a breakthrough Omicron infection. These findings reveal strong immunogenicity of a third vaccine in one of the most vulnerable population groups and endorse an approach for widespread delivery across this population. Ongoing assessment will be required to determine the stability of immune protection.

## Abstract (web summary)

Older people have suboptimal responses to primary series vaccines, which can place them at risk for adverse coronavirus disease 2019 outcomes. Here the authors show that booster vaccines provide a substantial increase in antibody levels in the short term but that there is significant waning 100 d after booster shots.

## Main

Age and frailty are major risk factors for severe coronavirus disease 2019 (COVID-19) outcome, and older residents of long-term care facilities (LTCFs) have suffered relatively high rates of mortality during the current pandemic^1^. Single or dual COVID-19 vaccination has provided strong clinical protection against severe disease within this group^2,3^ but there is concern about the potential impact of immune waning and the need for additional vaccines in those at greatest risk^4^. Severe acute respiratory syndrome coronavirus 2 (SARS-CoV-2) infection rates have been high in many LTCFs and studies have shown that many staff and residents have evidence of prior natural infection^5^. Importantly, this acts to strengthen vaccine-induced immunity such that older residents achieve comparable levels of antibody and cellular immunity as younger staff following dual primary series vaccination with mRNA or adenovirus-based vaccines^6,7^. However, these responses are markedly attenuated within the older adult population who have remained uninfected. In particular, antibody and cellular responses here are reduced by 62% and 50% respectively, compared to younger donors^4^. As such, the delivery of a third vaccine for this population has been prioritized.

The importance of boosting and sustaining vaccine-induced immune responses in older LTCF residents has been given considerable impetus through the emergence of the Omicron variant. This has a very high infection rate and evades a large component of the vaccine-induced humoral immune response^8–10^. In contrast, spike-specific cellular responses are more reliably maintained^11^, although these have not been assessed in older people. Third-dose vaccination appears effective in helping to suppress Omicron infection rates^12^, although it is not clear for how long this effect will be maintained due to antibody waning^13^.

We undertook an analysis of humoral and cellular spike-specific immune responses in staff and residents of LTCFs after the third vaccine dose and compared these to values that had been recorded after dual vaccination. We find that there is a robust antibody and cellular response to third vaccination in the older resident population, which is on par with the responses seen in the much younger population within LTCFs.

## Results

### Antibody responses are boosted strongly following third vaccination

Blood samples were obtained from 341 staff and residents within LTCFs following the third vaccine dose (Table 1). The median age of the staff was 48 years (interquartile range (IQR), 40–58 years, *n* = 183), while that of the residents was 84 years (IQR, 76–92 years, *n* = 158). Around 48% of donors received a primary series of mRNA vaccine (either BNT162b2 or mRNA-1273), while 52% received ChAdOx1. The third vaccine comprised an mRNA formulation in every case with 336/341 recipients receiving BNT162b2 (Pfizer) and 5/341 recipients receiving mRNA-1273 (Moderna). Residents received their third vaccine dose somewhat earlier than staff and samples were obtained at a median of 92 d (IQR, 31–113 d) following the third vaccine.

**Table 1:** Donor demographics

	Staff	Resident	Total	% of total
**Total third vaccine participants (*n*)**	183	158	341	100
**Age ≥80 years**	0	107 (69+)	107 (69+)	31 (65+)
**Age 65–79 years**	8 (5+)	43 (27+)	51 (32+)	15 (62.7+)
**Age** ≤**64 years**	175 (94+)	8 (3+)	183 (97+)	54 (53+)
**Median age in years (IQR)**	48(40–58)	84(76–92)	63(48–85)	
**Female**	164 (91+)	103 (69+)	267 (160+)	78.3 (60+)
**Male**	19 (8+)	55 (30+)	74 (38+)	21.7 (51+)
**Baseline mRNA vaccine recipients**	99 (52+)	64 (46+)	163 (98+)	47.8 (60.1+)
**Baseline ChAdOx1 recipients**	84 (47+)	94 (53+)	178 (100+)	52.2 (56.2+)
**Median days between third vaccine and sample (IQR)**	93 (38–113)	77 (25–113)	92 (31–113)	
**Total LTCFs**	48			
**Mean participants/LTCF (s.d.)**	7.1 (5.1)			

+ indicates SARS-CoV-2 prior infection.

Spike-specific and nucleocapsid-specific antibody levels were determined using the MSD platform. A positive nucleocapsid-specific value or prior history of PCR-confirmed COVID-19 infection before sampling was taken as evidence of prior natural infection and subsequent studies were analyzed in relation to infection status.

**Fig. 1:**
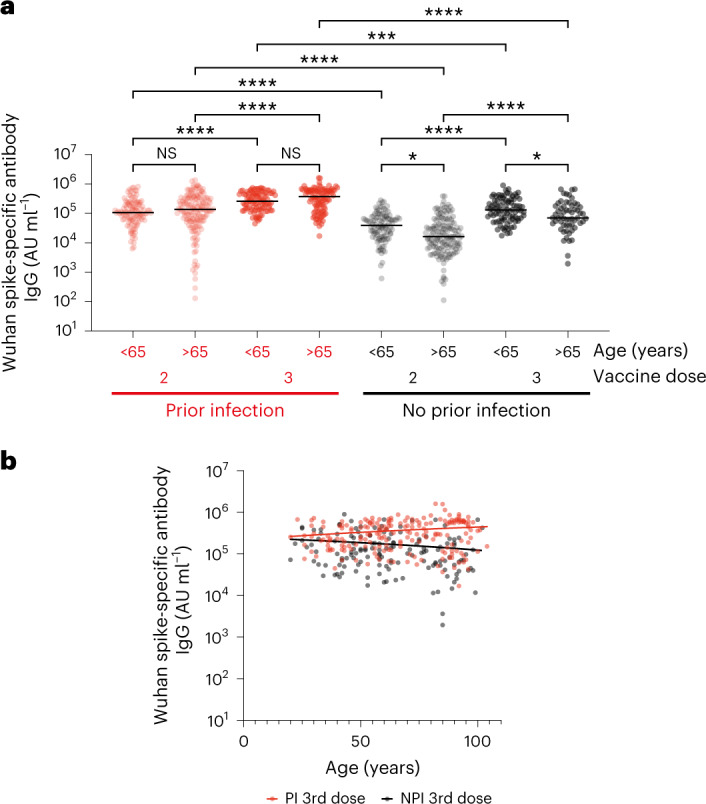
Spike-specific antibody responses are boosted after third vaccine. **a**, Wuhan spike-specific antibody titer after 2 or 3 COVID-19 vaccine doses in relation to prior infection status and in staff or resident groups. Red dots indicate participants with prior natural infection and black dots indicate non-infected donors. Age is shown as <65 or >65 years and separates staff and residents. Kruskal–Wallis (uncorrected Dunn’s test), *P = 0.05, ****P < 0.0001, *n* = 863. The black line indicates the median antibody titer. **b**, Spike-specific antibody titer in relation to donor age. Red dots indicate participants with prior natural infection (PI; Spearman’s correlation *r* = 0.13, *P* = 0.053, *n* = 198) and black dots indicate those with no prior infection (NPI; two-tailed Spearman’s correlation *r* = −0.21, *P* = 0.01, *n* = 145). All fitted lines are linear regressions.

Initial analyses compared antibody levels in donors aged <65 and >65 years, which segregated >95% of staff and residents by age. Antibody levels in both staff (<65 years) and residents (>65 years) increased strongly following the third vaccine. Among those with prior infection, titers increased by 2.4-fold (106,700 arbitrary units per milliliter (AU ml^−1^; 961 binding antibody units per milliliter (BAU ml^−1^)) versus 259,327 AU ml^−1^ (2,336 BAU ml^−1^)) and 2.7-fold (136,898 AU ml^−1^ (1,233 BAU ml^−1^) versus 374,158 AU ml^−1^ (3,371 BAU ml^−1^); *P* < 0.0001) in staff and residents compared to dual vaccination, respectively^4^. This increment was more marked in infection-naive individuals where values increased by 3.3-fold (39,149 AU ml^−1^ (352 BAU ml^−1^) versus 131,127 AU ml^−1^ (1,181 BAU ml^−1^)) and 4.3-fold (16,299 (146 BAU ml^−1^) AU ml^−1^ versus 70,522 AU ml^−1^ (635 BAU ml^−1^); *P* < 0.0001), respectively (Fig. 1a). Despite this increment, antibody titers in infection-naïve older residents remained 42% lower than younger staff members (131,127 AU ml^−1^ (1,181 BAU ml^−1^) versus 70,522 AU ml^−1^ (635 BAU ml^−1^); *P* = 0.05). Of note, prior infection with SARS-CoV-2 consistently boosted antibody responses across the life course even after the third vaccine and this was modestly increased in those with a Charlson Comorbidity Index (CCI) score of 3+ (Supplementary Fig. 1). Of note, the baseline primary series vaccine regimen had no substantial impact on the humoral response following third-dose vaccine (Supplementary Fig. 2).

The impact of age and natural infection on spike-specific antibody response was next assessed using age as a continuous variable (Fig. 1b). A trend was seen toward a higher-level response in older people with prior infection but this was not significant (*r* = 0.13, *P* = 0.053).

These data show that third vaccine doses are effective in augmenting antibody levels beyond those seen following the primary series and that this increment is particularly marked in the older adult cohort.

### Antibody responses following third vaccine show pronounced waning

**Fig. 2:**
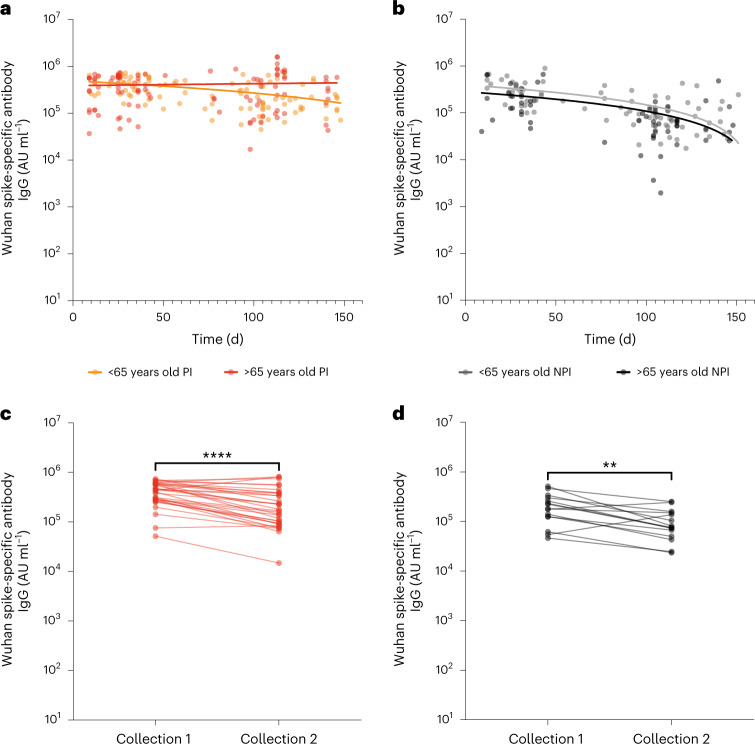
Spike-specific antibody responses show pronounced waning after third dose. **a**, Spike-specific antibody titer in relation to the day after the third vaccine in donors with prior infection. Orange indicates staff (Spearman’s correlation *r* = −0.48, *P* < 0.0001, *n* = 97) and red indicates residents (two-tailed Spearman’s correlation *r* = 0.003, *P* = 0.97, *n* = 101). All fitted lines are linear regressions. **b**, Spike-specific antibody titers in relation to the day after the third vaccine in donors without prior infection. Gray indicates staff (Spearman’s correlation *r* = −0.63, *P* < 0.0001, *n* = 86) and black indicates residents (two-tailed Spearman’s correlation *r* = −0.50, *P* < 0.0001, *n* = 57). All fitted lines are linear regressions. **c**, Spike-specific antibody titer in individual paired samples at two time points following third vaccine dose in donors with prior infection. Two-tailed paired *t*-test *P* < 0.0001, *n* = 32. **d**, Spike-specific antibody titers in individual paired samples at two time points following third vaccine dose in donors without prior infection. Two-tailed paired *t*-test *P* < 0.0044, *n* = 15.

We next assessed spike-specific antibody levels in relation to time after third vaccine delivery to assess for potential immune waning. Median values within the first 50 d after vaccination were taken as the peak response and were compared to those at 100–150 d. Antibody levels were seen to fall markedly during this 100-d period and this was influenced by both prior infection status and age. In particular, median titers fell by 62% and 21% in <65 year olds and >65 year olds with a previous SARS-CoV-2 infection, respectively, compared to 78% and 75% in those who were infection-naive, respectively. Spearman’s rank correlation analyses confirmed the stability of immune response in prior infected older people (*r* = 0.003, *P* = 0.97), although a decline was observed in staff (*r* = −0.48, *P* < 0.0001; Fig. 2a). A strong statistical association with antibody waning over time was observed in staff and residents who remained infection-naive (*r* = −0.63, *P* = < 0.0001 and *r* = −0.50, *P* < 0.0001, respectively; Fig. 2b).

To further assess the importance of antibody waning, we also utilized access to 47 pairs of serum samples that were taken at different time points from individual donors after the third vaccine dose. The median time between samples was 81 d (IQR, 79–105 d) in donors with prior infection and antibody levels fell by 60% (438,433 AU ml^−1^ (3,950 BAU ml^−1^) versus 174,066 AU ml^−1^ (1,568 BAU ml^−1^); Fig. 2c). Titers fell by 58% (179,705 AU ml^−1^ (1,619 BAU ml^−1^) versus 75,916 AU ml^−1^ (684 BAU ml^−1^)) within the infection-naive group (median time between samples 77 d, (IQR, 73–83 d); Fig. 2d).

As such, third vaccine doses elicited strong peak antibody responses irrespective of age or infection status. However, these values were not sustained and fell substantially within 3 months in all groups except older people with prior infection.

Antibody binding to the whole spike or receptor-binding domain (RBD) of the Wuhan, Delta and Omicron viral variants was also assessed on MSD plates in participants who received a booster vaccine. (Supplementary Fig. 3). Antibody binding was strongest against the Wuhan spike protein but reduced by 40% (*P* < 0.0001) and 52% (*P* < 0.0001) against Delta in donors with prior infection or who were infection-naive, respectively, whereas values were 76% (*P* < 0.0001) and 75% (*P* < 0.0001) lower against Omicron, respectively. Binding to the RBD of Wuhan and Delta was equivalent but a marked 81% (*P* < 0.0001) and 78% (*P* < 0.0001) fall in binding to Omicron was seen in prior infected or infection-naive cohorts. These findings show that relative antibody binding to Omicron, in particular the RBD domain, is markedly reduced after three vaccine doses.

### Neutralization of variants improved following third vaccine dose

Viral neutralization is emerging as a valuable immune correlate of protection and the ability of post-vaccine serum to neutralize live virus was next determined. Samples after the second and third vaccine doses were assessed for neutralization of Delta and Omicron variants and assessed in relation to prior infection status and age.

**Fig. 3:**
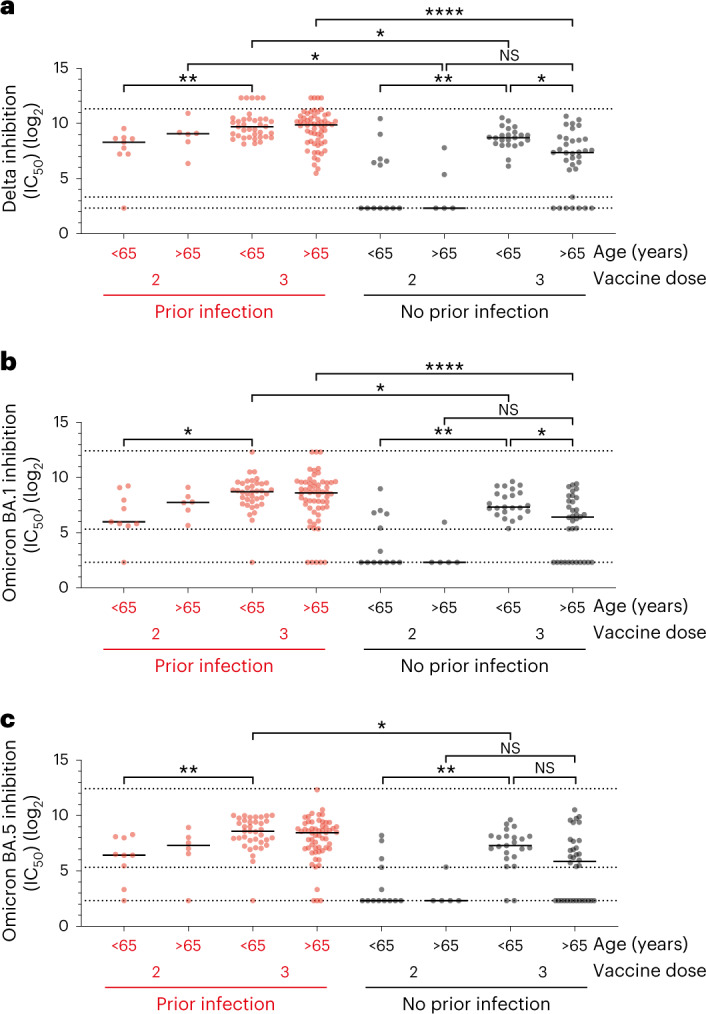
Third vaccine dose enhances neutralization of Delta and Omicron BA.1 and BA.5. **a**, Antibody neutralization of Delta variant after third vaccine in relation to prior natural infection and age. Red dots indicate participants with prior natural infection and black dots indicate non-infected donors. Kruskal–Wallis (uncorrected Dunn’s test), *P = 0.015, **P = 0.003, ****P = 0.0001, *n* = 195. Black line indicates the median. No prior infection <65 versus >65 years old, Chi-squared test: Fisher’s exact, *P* = 1.000. Top and bottom dotted lines indicate the continuous region of the data obtained. Between middle and bottom regions is the weak inhibition region. **b**, Antibody neutralization of Omicron variant BA.1 after third vaccine in relation to prior natural infection and age. Red dots indicate participants with prior natural infection and black dots indicate non-infected donors. Kruskal–Wallis (uncorrected Dunn’s test), *P = 0.03, **P = 0.003, ****P = 0.0001, *n* = 195. Black line indicates the median. No prior infection <65 versus >65 years old, Chi-squared test: Fisher’s exact, *P* = 0.596. Top and bottom dotted lines indicate the continuous region of the data obtained. Between middle and bottom regions is the weak inhibition region. **c**, Antibody neutralization of Omicron variant BA.5 after third vaccine in relation to prior natural infection and age. Red dots indicate participants with prior natural infection and black dots indicate non-infected donors. Kruskal–Wallis (uncorrected Dunn’s test) *P = 0.01, **P = 0.004, *n* = 195. Black line indicates the median. No prior infection <65 versus >65 years old, Chi-squared test: Fisher’s exact, *P* = 0.46. Top and bottom dotted lines indicate the continuous region of the data obtained. Between middle and bottom regions is the weak inhibition region.

In donors with prior infection, the neutralization of Delta was robust after the second and third vaccines, although a 2.6-fold (314 versus 830 IC_50_ value; Fig. 3a, *P* = 0.003) increment was observed in younger donors with third vaccine. In contrast, infection-naive donors required a third vaccine to generate substantial neutralization, although this remained attenuated in the resident population with a 2.5-fold (418 versus 163 IC_50_) reduction compared to staff (*P* = 0.015). Of note, a similar profile was seen in relation to neutralization of the Omicron BA.1 and BA.5 variants, although all values were at a lower level (Fig. 3b,c and Supplementary Fig. 4).

These observations indicate that, in the absence of prior infection, a primary series dual vaccination is unlikely to provide sufficient sustained protection against either Delta or Omicron infection, but neutralization is enhanced after three vaccines, particularly in younger donors.

### Antibody binding against Wuhan spike protein correlates strongly with neutralization

**Fig. 4:**
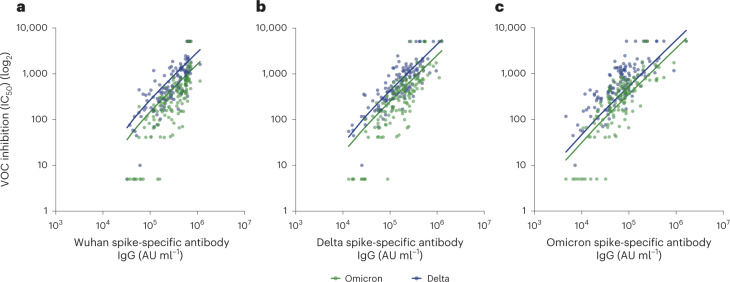
Spike-specific antibody binding to Wuhan spike protein correlates with variant neutralization. **a**, Wuhan spike-specific antibody titers in relation to variant of concern (VOC) neutralization. Blue dots and line indicate Delta neutralization (two-tailed Spearman’s correlation *r* = 0.80, *P* < 0.0001, *n* = 133) and green dots and line indicate Omicron neutralization (two-tailed Spearman’s correlation *r* = 0.82, *P* < 0.0001, *n* = 133). All fitted lines are linear regressions. **b**, Delta spike-specific antibody titer in relation to VOC neutralization. Blue dots and line indicate Delta neutralization (two-tailed Spearman’s correlation *r* = 0.81, *P* < 0.0001, *n* = 133) and green dots and line indicate Omicron neutralization (two-tailed Spearman’s correlation *r* = 0.81, *P* < 0.0001, *n* = 133). All fitted lines are linear regressions. **c**, Omicron spike-specific antibody titer in relation to VOC neutralization. Blue dots and line indicate delta neutralization (two-tailed Spearman’s correlation *r* = 0.76, *P* < 0.0001, *n* = 133) and green dots and line indicate omicron neutralization (two-tailed Spearman’s correlation *r* = 0.80, *P* < 0.0001, *n* = 133). All fitted lines are linear regressions.

An immune correlate of protection that is measurable within the population could help to guide assessment of personalized risk of infection. Viral neutralization assays are not feasible for clinical use and as such it is important to understand how antibody titer correlates with neutralization activity. We next correlated the magnitude of antibody binding within post-vaccine sera against spike protein from each of Wuhan, Delta and Omicron BA.1 with the degree of neutralization of the Delta and Omicron variants (Fig. 4a–c).

Importantly, binding to spike proteins from Wuhan, Delta and Omicron was strongly correlated with neutralization of both Delta and Omicron (*P* < 0.001 for each of the three correlations). Furthermore, the strength of the correlation was broadly equivalent in each case (*r* = 0.77–0.83) and was strongest for the correlation of Wuhan spike binding and Omicron neutralization.

These findings show that assays measuring vaccine-induced antibody binding to Wuhan spike are a correlative marker for neutralization of the current dominant Omicron variant.

Importantly, 22/195 (11%) of donors showed no neutralization activity against Delta despite a spike-specific immune response. This group were all infection-naive and their median spike-specific antibody response was 3,519 AU ml^−1^ (IQR, 768–12,025; Supplementary Fig. 5) compared to 300,789 AU ml^−1^ (IQR, 142,710–550,512) in those with neutralization activity. Selection of an antibody level that predicts neutralization is important as choosing an inappropriately low threshold could lead to vulnerable individuals being denied additional vaccine doses or therapeutic monoclonal antibodies. These data indicate that an MSD value > 142,710 AU ml^−1^ (1,285 BAU ml^−1^) would identify all nonresponders with >70% specificity.

### Strong, stable and cross-reactive cellular responses are induced following third vaccination

Cellular responses are increasingly recognized as key mediators of immune protection with a central role in protection against viral variants and severe infection. Thus, we next assessed spike-cellular responses in LTCF donors using the interferon (IFN)-γ ELISpot assay.

**Fig. 5:**
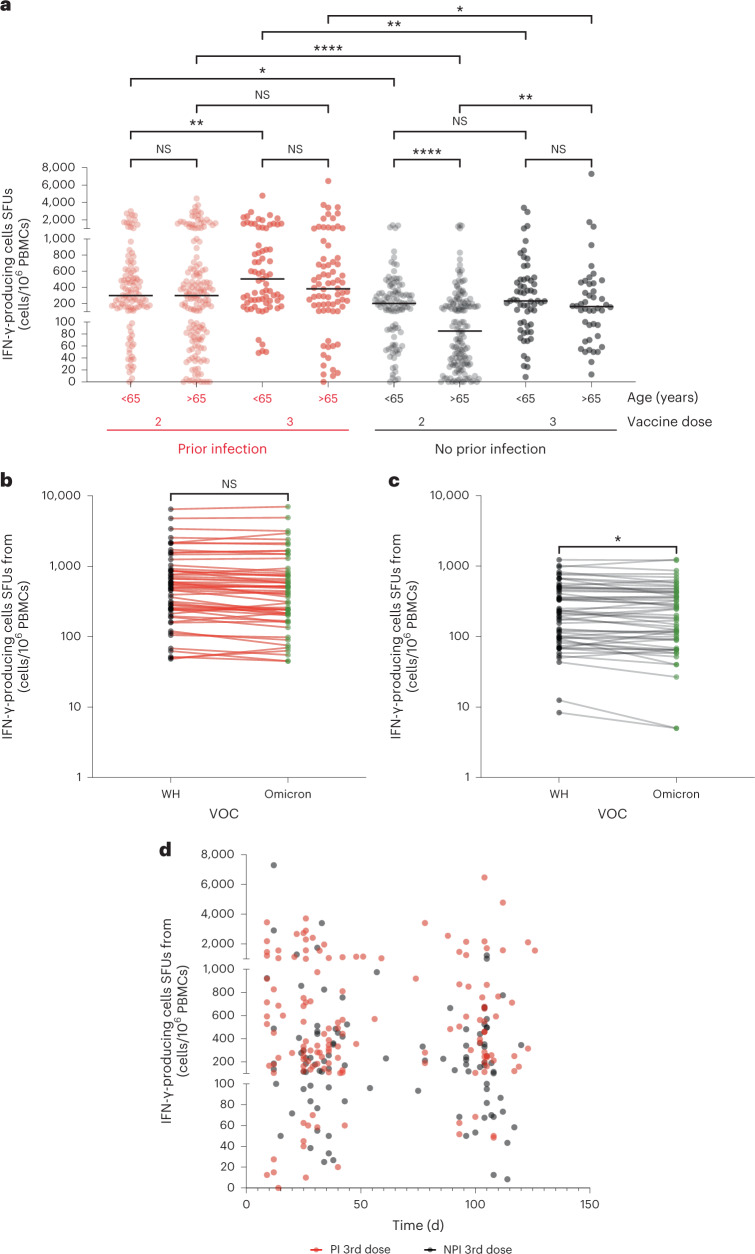
Spike-specific T cell responses are increased following third vaccination. **a**, IFN-γ ELISpot response to spike peptide stimulation following second or third vaccine. Results are shown in relation to age (<65 and >65 years), prior natural infection (red) and no prior infection (black). Kruskal–Wallis (uncorrected Dunn’s test) *P = 0.01, **P = 0.009, ****P < 0.0001, *n* = 768. Black lines indicate the median. **b**, Paired IFN-γ ELISpot responses in individual donors following stimulation of PBMCs with peptides from Wuhan spike (black) and Omicron spike (green) in prior infected donors. Two-tailed paired *t*-test *P* = 0.34, NS, *n* = 57. **c**, Paired IFN-γ ELISpot responses in individual donors following stimulation of PBMCs with peptides from Wuhan spike (black) and Omicron spike (green) in infection-naive donors. Two-tailed paired *t*-test *P = 0.02, *n* = 57. **d**, IFN-γ ELISpot response to spike peptide stimulation in relation to the day after the third vaccine. Red indicates participants with prior natural infection (two-tailed Spearman’s correlation *r* = 0.04, *P* = 0.66, *n* = 145) and black indicates infection-naïve donors (two-tailed Spearman’s correlation *r* = −0.12, *P* = 0.22, *n* = 103).

As previously reported^4^, cellular responses were robust after two vaccines in participants with prior infection, although this increased a further 1.7-fold (300 spot forming units (SFUs)/10^6^ peripheral blood mononuclear cells (PBMCs) versus 505 SFUs/10^6^ PBMCs) after a third vaccine within those under 65 years (Fig. 5a; *P* = 0.0098). No differences in cellular response between donors over and under 65 years old were seen in this subgroup of prior infected individuals. In contrast, in the absence of prior infection cellular responses within those over 65 years old were markedly inferior by 2.4-fold (203 SFUs/10^6^ PBMCs versus 85 SFUs/10^6^ PBMCs) to those under 65 years old after second vaccination (*P* < 0.0001) indicating potential vulnerability to impaired clinical outcome. Cellular responses were boosted by 1.1-fold (203 SFUs/10^6^ PBMCs versus 233 FUs/10^6^ PBMCs (*P* = 0.19) in those under 65 years and an encouraging 1.9-fold (85 FUs/10^6^ PBMCs versus 162 FUs/10^6^ PBMCs; *P* = 0.029) in those over 65 years old after the third vaccine. The preferential increment in older donors was such that responses were equivalent across both groups.

Comparative cellular responses against peptides from the Wuhan and Omicron spike proteins were then assessed. Importantly, paired analysis showed no difference in the cellular response against Wuhan and Omicron in donors with prior infection after three vaccines (Fig. 5b). Within infection-naive donors, the cellular response against Omicron was 92% (218 FUs/10^6^ PBMCs versus 200 FUs/10^6^ PBMCs) of that observed against the Wuhan spike pool (Fig. 5c; *P* = 0.027). No differences in these responses were observed in relation to donor age. Cellular responses against the S1 domain of each spike subtype were dominant over S2 (Supplementary Fig. 6). A strong association between virus neutralization and cellular response was also observed indicating coordinated humoral and cellular immunity (Delta, *r* = 0.32, *P* < 0.0001; Omicron, *r* = 0.33, *P* < 0.0001; Supplementary Fig. 7).

Given that noteable antibody waning was observed within the first 100 d after the third vaccine, we next assessed potential waning of the cellular response. Importantly, in both prior infected and infection-naive donors, we did not observe waning in the cellular response (Fig. 5; prior infection, *r* = 0.04, *P* = 0.66; no prior infection, *r* = −0.12, *P* = 0.22). Of note, the baseline primary series vaccine regimen had no impact on the cellular response following the third vaccine dose (Supplementary Fig. 8)

These findings show that cellular responses are consistently boosted after three vaccines and become equivalent between younger and older donors. Furthermore, the great majority of this response is retained against the Omicron variant, implying strong residual protection during the current pandemic, and no short-term waning of response is observed.

### Spike-specific CD4^+^ T cells are dominated by IL-2 expression

To assess the features of spike-specific T cells in more depth, we used intracellular cytokine analysis to identify and phenotype virus-specific populations. CD4^+^ T cells dominated the global T cell response and the magnitude of the CD8^+^ populations was too low to reliably facilitate further analysis.

**Fig. 6:**
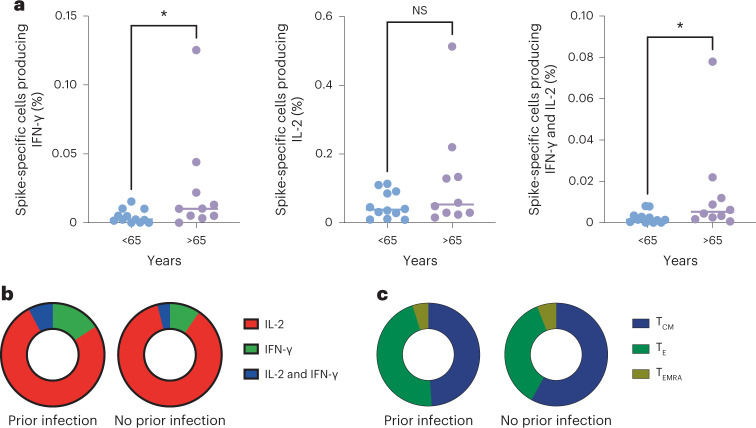
Frequency and phenotype of spike-specific CD4^+^ T cells. **a**, Frequency of spike-specific CD4^+^ T cells within the total CD4^+^ repertoire as detected by intracellular cytokine analysis and in relation to single or dual production of IL-2 or IFN-γ. The *x* axis indicates donor age (<65 or >65 years). Two-tailed Mann–Whitney, IFN-γ *P = 0.025, IL-2^+^IFN-γ^+^ *P = 0.017. **b**, Pattern of single or dual IL-2 and IFN-γ cytokine production in spike-specific CD4^+^ T cells in donors with prior infection (*n* = 13) or no prior infection (*n* = 9). (PI: IL-2, IFN-γ and IL-2^+^IFN-γ^+ ^= 76%, 16% and 8%, versus NPI 86%, 10% and 4%, respectively). Two-tailed Chi-square *P* < 0.0001 PI versus NPI. **c**, Distribution of central memory (T_CM_), effector (T_E_) or T_EMRA_ memory pools in spike-specific CD4^+^ T cells in donors with prior infection (*n* = 13) or no prior infection (*n* = 9). (PI, T_CM_, T_E_ and T_EMRA _= 49%, 46% and 5%, versus NPI = 58%, 36% and 6%, respectively). Two-tailed Chi-square *P* = 0.36, PI versus NPI.

Virus-specific CD4^+^ cells comprised populations that secreted single or dual expression of interleukin (IL)-2 and IFN-γ (Fig. 6a). Indeed, single-positive IL-2 cells were the most common, representing 8.2 per 10,000 CD4^+^ cells (0.082%). This was 4.1-fold higher than the pool of cells that secreted IFN-γ and indicates that IFN-γ-based assays such as ELISpot may considerably underestimate the memory pool. About 65% of IFN-γ-positive cells were single positive, while 35% had a dual IL-2^+^IFN-γ^+^ phenotype. Stratification of these virus-specific cells by cytokine profile revealed that prior infection increased the proportion of IFN-γ^+^ cells from 14% within infection-naive donors to 24% in those with prior infection (Fig. 6b). Analysis of memory phenotype showed that virus-specific responses were dominated by central memory and effector-memory populations (Fig. 6c).

Finally, the relationship between memory subset, cytokine production and differentiation status as assessed by CD27 and CD28 coexpression was determined (Supplementary Fig. 9). This showed loss of CD27^−^ in 25–50% of cells during transition to the effector pool and accumulation of a CD27^−^CD28^−^ subset within the effector-memory CD45RA-revertant (TEMRA) pool. IFN-γ^+^ expression was markedly enhanced within the highly differentiated pool.

### Spike-specific immune responses do not correlate with protection against Omicron infection

Initial third vaccine doses within this cohort largely predated the Omicron variant but national booster administration programs were accelerated with the aim of reducing its impact. As such, we next determined the rate of breakthrough infection within the whole cohort and assessed the potential influence of the magnitude of spike-specific antibody or cellular responses on the risk of infection.

**Fig. 7:**
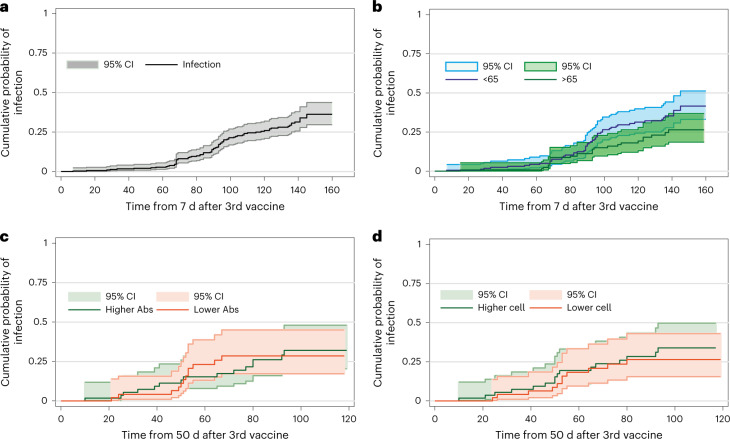
Peak antibody and cellular response after third vaccine do not correlate with protection against breakthrough. **a**, Kaplan–Meier curve showing the probability of infection from third vaccine in recipients who had follow-up screening after vaccination. Follow-up time began 7 d after the third vaccine (*n* = 305, 81 positive PCR or LFD). The 95% confidence intervals (CIs) are shown in a lighter shade. **b**, Kaplan–Meier curve showing the probability of infection from third vaccine in recipients stratified by <65 and ≥65 years of age. Follow-up time began 7 d after the third vaccine (blue, <65 years, 54 positive test; green, ≥65 years, 27 positive test, *n* = 305, log-rank *P* = 0.099). The 95% CIs are shown in a lighter shade. **c**, Kaplan–Meier curve of the probability of infection in relation to spike-specific antibody response above predicted (green) or below predicted (red) median over the subsequent 120 d. Cox proportional hazards regression model: hazard ratio, 0.96 (95% CI 0.45–2.06, *P* = 0.93), *n* = 122 participants. **d**, Kaplan–Meier curve of the probability of infection in relation to spike-specific cellular response above (green) or below (red) predicted median over the subsequent 120 d. Cox proportional hazards regression model: hazard ratio, 0.81 (95% CI 0.37–1.74, *P* = 0.59), *n* = 122 participants.

Donors were followed from 7 d after the date of the third vaccine and 305 were identified who had PCR or lateral flow device (LFD) tests undertaken after vaccine. In total, 81 (27%) developed infection during follow-up of 170 d (4,822 person-days; Fig. 7a). Only one donor was hospitalized and there were no fatal outcomes. Breakdown by age showed no difference in infection rate in relation to age <65 and ≥65 years (*P* = 0.099; Fig. 7b).

Data on spike-specific antibody and cellular immune response within the 7–50-d period of peak response after the third vaccine were available for 122 participants. Comparison of donors with antibody or cellular responses that were below or above the median predicted value showed no relationship to risk of infection over the subsequent 120 d (Fig. 7c,d).

## Discussion

The COVID-19 pandemic is estimated to have led to the death of over 18 million people and older people who require residential and/or nursing care are at particularly high risk of mortality. Vaccines have transformed the clinical outlook but many questions remain regarding their optimal delivery. Here we undertook a detailed assessment of the efficacy of a third vaccine dose within this age group and identified a number of features that can help to guide future vaccine policy.

At the time of the introduction of COVID-19 vaccines, the primary series course consisted of either one or two vaccine doses and there was hope this might provide long-term protection. However, the uncovering of immune waning and breakthrough infection has led to recommendations that additional vaccine doses should be administered to many demographic groups^4,14–16^. For healthy immunocompetent people, these additional doses are typically termed ‘booster vaccines’, while for those with immune suppression they have become regarded as a constituent of the primary series^17^. The third vaccine is generally regarded as a booster vaccine in older residents of care homes despite that this group is typically relatively immunosuppressed due to age and comorbidity^18,19^. As such, within this report we refer to the third vaccine dose without reference to its primary or booster status.

Previous studies have revealed a deficit in immune protection within older people in residential care following COVID-19 vaccination^20–22^. A relatively unique feature of early studies of vaccine immunogenicity in care homes was the high rates of prior natural infection within this community^23^. As such, this allowed assessment of the impact of previous SARS-CoV-2 infection on vaccine immunogenicity. Indeed, infection substantially increased both the magnitude and functional quality of the antibody and cellular response^24^. However, a deficit in immune response was seen within infection-naive residents following primary series vaccination with lower levels of antibody and cellular response compared to younger staff members^4,16^. This provides the basis for the current assessment.

Third vaccine doses strongly improved vaccine immunogenicity. In particular, antibody levels increased by between 2.4-fold and 4.3-fold when assessed by prior infection status or age. An incremental, albeit smaller, improvement was also observed in the cellular response. An important and encouraging feature was that immune responses were enhanced in older people without prior infection, the group with the greatest prior deficit.

The Omicron SARS-CoV-2 variant is now globally dominant and it is essential to assess vaccine-induced immunity against this challenge. Overall findings are encouraging. Antibody binding to Omicron spike and RBD was lower than against the original Wuhan strain. The level of Omicron BA.1/BA.5 neutralization was also lower compared to Delta. However, a third vaccine was seen to be essential to induce neutralization activity with a increase in neutralization after booster observed in younger donors (<65 years). A similar trend was seen in those >65 years old, although statistical significance was not reached. This may partially reflect an age-related effect but may also relate to lower sample numbers. Cellular responses were also extremely well maintained against Omicron with a fall of only 8–12% compared to recognition of peptide pools from Wuhan spike^25,26^. These findings are compatible with epidemiological studies of Omicron infection within care homes, which show a markedly reduced rate of disease severity compared to previous waves of infection^12^.

A feature of our study that raises some concern for future protection was the rate of decline of spike-specific antibodies following the third vaccine dose. This fell by 21–78% within different subgroups and was notably higher in those without prior infection. Furthermore, waning was somewhat higher in staff, a feature previously observed for the nucleocapsid-specific response^14^, and may reflect relative selection for older survivors with a more robust immune system. In contrast, cellular responses remained stable. Further assessment of this cohort is required to assess the longer-term stability of antibody responses and clinical protection. However, these findings will contribute to suggestions for the need of a fourth vaccine dose, particularly given somewhat suboptimal neutralization responses in the resident population. Initial studies of a fourth vaccine within younger donors indicate that peak immune responses may exceed those seen after the third vaccine^27^.

The development of immune correlates of protection could transform the introduction of new and optimized vaccines. Antibody magnitude and relative neutralization activity are two such factors, although neutralization assays remain challenging to determine. Taking advantage of our large dataset, we were able to demonstrate that antibody binding to the ancestral Wuhan spike is strongly correlated with neutralization of all tested viral variants, including Omicron. As such, this provides support for the use of this determinant as a potential measure of personal protection against subsequent infection.

Considerable debate is being given to the potential development of novel vaccine formulations that may act to sustain vaccine-induced immune responses in the longer term. As such, increasing attention is being focused on detailed features of the adaptive immune response. CD4^+^ T cell responses were found to dominate the cellular responses and IL-2^+^ cells were eightfold higher than those producing IFN-γ, indicating that IFN-γ ELISpot assays are likely to underestimate virus-specific cellular memory. Similar features have been observed after natural infection^28^ and these high levels of IL-2 production augur well for potential long-term immune memory. Indeed, many spike-specific CD4^+^ T cells remained within the central memory subset that replenishes effector pools. Natural infection increased the proportion of IFN-γ effector cells, and the relationship of this observation to improved clinical protection will be an important area for future study. In contrast, a late-differentiated CD27^−^CD28^−^ pool emerged within the T_EMRA_ subset and this phenotype is associated with reduced proliferative potential^29^. The extent to which repeated vaccine dosing may lead to ‘vaccine exhaustion’ within spike-specific T cell populations has not yet been assessed, but these features indicate that this should now be addressed, particularly in older populations in whom naive T cell pools are limited.

Finally, we also assessed how the magnitude of the peak immune response after the third vaccine dose might relate to protection against breakthrough infection. Roughly 27% of participants developed infection over the 170 d of follow-up and this rate is consistent with the high prevalence of Omicron variant over this period. Immune response data of the peak response within the first 50 d after vaccine were available from 122 participants but peak response was not associated with risk of infection. However, the clinical severity of infection was modest, with only one hospitalization, and this is likely to reflect the strong impact of vaccine-induced immune protection. As such, a larger epidemiological study is required to study the clinical impact of booster infection on protection against severe disease.

There are limitations of this study. We did not have access to the exact time or severity of primary infection for participants with prior infection. Information on patient ethnicity was also not available. There is also the potential for waning of nucleocapsid-specific IgG such that prior infected donors may be missed, although we estimate this risk as low. Asymptomatic infections may also have occurred following serological assessment of serostatus and these factors may limit interpretation of immune protection correlates. Furthermore, this was a retrospective analysis of prospectively collected data.

In conclusion, we show that a third vaccine dose is highly effective at boosting antibody and cellular responses in older and vulnerable residents in care homes and is essential to deliver antibody and cellular protection against Omicron. Thus, we suggest that these should be regarded as a constituent to primary series vaccination in this vulnerable cohort. However, rapid antibody waning was observed in infection-naive individuals and breakthrough infections occurred in all groups. Further studies are needed to assess the potential value of additional vaccine doses.

## Methods

### Sample collection

The VIVALDI study (ISRCTN 14447421) is a prospective cohort study, which was set up to investigate SARS-CoV-2 transmission, infection outcomes and immunity in residents and staff in LTCFs in England that provide residential and/or nursing care for adults aged 65 years and over (https://wellcomeopenresearch.org/articles/5-232/v2/).

Eligible LTCFs were identified by the care provider’s senior management team, or by the National Institute for Health Research Clinical Research Network. Pseudonymized clinical data (vaccination status, PCR test results, hospitalization, death) and demographic data (age, sex, staff member versus resident) were retrieved for staff and residents from participating LTCFs through national surveillance systems. All participants provided written informed consent for blood sample collection or if residents lacked the capacity to consent, a personal or nominated consultee was identified to act on their behalf.

Blood sampling was carried out from 25 May 2021 until 23 of February 2022. An anti-coagulated EDTA blood sample was sent to the University of Birmingham and a serum tube was also obtained for The Doctors Laboratory where anti-nucleocapsid IgG (N) testing using the Abbott immunoassay was performed as well as a viral neutralization assay at the Crick Institute. Ethical approval for this study was obtained from the South Central-Hampshire B Research Ethics Committee (20/SC/0238).

### Data linkage

Abbott antibody test results were submitted to the COVID-19 data store (https://data.england.nhs.uk/covid-19/), pseudonymized and linked to routinely held data on age, sex, LTCF, role (staff or resident) and results of PCR or LFD SARS-CoV-2 testing performed through the national SARS-CoV-2 testing program. Asymptomatic screening using PCR tests was performed weekly in staff and monthly in residents with more frequent LFD or PCR testing during outbreaks. Using the common pseudo-identifier based on the individuals’ National Health Service (NHS) number, linkage was undertaken to vaccination status (date and vaccine type) derived from the National Immunisation Management System and dates and diagnostic codes for hospitalizations recorded in the Hospital Episode Statistics dataset as well as for any deaths from the Office for National Statistics dataset. Individual-level records were further linked to each LTCF using the unique Care Quality Commission location ID, allocated by the Care Quality Commission, who regulate all providers of health and social care in the United Kingdom.

### Inclusion criteria

Both staff and residents were eligible for inclusion if samples could be linked to a pseudo-identifier enabling data linkage. We included samples from participants who had received a primary vaccine course with or without a third vaccine dose. Participants sampled in the 7 d following the third vaccine administration were excluded from the analysis to ensure peak immune responses were reached before sampling. Due to limited PCR testing in the first wave of the pandemic, it was not possible to determine when individuals had been infected with SARS-CoV-2 based on PCR alone. Previous infection with SARS-CoV-2 was defined based on results of the MSD antibody test and Abbott’s test using thresholds and methods outlined below. No statistical methods were used to predetermine sample sizes but our sample sizes are similar to those reported in previous publications^5,14,16,28^.

### Sample preparation

Samples were processed within 24 h of reception at the University of Birmingham. Blood was spun at 300*g* for 5 min. Plasma was removed and spun at 500*g* for 10 min before storage at −80 °C. The remaining blood was separated using a SepMate (Stemcell) density centrifugation tube. The resulting PBMC layer was washed twice with RPMI and rested overnight in R10 (RPMI + 10% FBS + penicillin–streptomycin) medium at 37 °C in 5% CO_2_.

### Serological analysis of SARS-CoV-2-specific immune response

Quantitative IgG antibody titers were measured against trimeric spike (S) protein and nucleocapsid (N) protein and VOCs using the MSD V-PLEX COVID-19 IgG Kit (SARS-CoV-2 panels 2, 22 and 23) following manufacturer’s instructions (K0081795). Briefly, 96-well plates were blocked. Following washing, plasma samples were diluted at a 1:5,000 ratio in diluent and added to the wells with the reference standard and internal controls. After incubation, plates were washed and anti-IgG detection antibodies were added. Plates were washed and were immediately read using a MESO TM QuickPlex SQ 120 system. Data were generated by Methodical Mind software and analyzed with MSD Discovery Workbench (v4.0) software. Presented data were adjusted for any sample dilutions.

### Quantification of SARS-CoV-2-specific cellular responses by ELISpot

The PepMix pool containing 15-mer peptides overlapping by ten amino acids from either SARS-CoV-2 Wuhan or Omicron spike S1 or S2 protein domains were purchased from JPT Peptide Technologies. T cell responses of post-vaccination and post-booster samples to the above peptide mixes were determined using a Human IFN-γ ELISpot PRO kit (Mabtech). Isolated PBMCs rested overnight in R10 (RPMI + 10% FBS + penicillin–streptomycin) at a concentration of 2–3 × 10^5^ cells were stimulated in duplicate with peptide mixes at 2 μg ml^−1^ for each peptide, anti-CD3 and CEFX cell stimulation mix (JPT, PM-CEFX-2) as a positive control, or dimethylsulfoxide as a negative control for 16–18 h. Supernatants were harvested and stored at −80 °C. Following the development of plates according to the manufacturer’s instructions, the plates were read using the BioSys Bioreader 5000. Mean spot counts in dimethylsulfoxide-treated negative control wells were deducted from the means to generate normalized spot counts for all other treated wells. Cutoff values were previously determined^16^.

### Intracellular cytokine staining

Around 1.5 x 10^6^ PBMCs were stimulated with either SARS-CoV-2 spike S1 or S2 peptide pool at a final concentration of 2 ng ml^−1^ per peptide for 6 h. Protein transport inhibitor and CD107a-specific antibody were added after 1 h and PBMCs were washed with MACS (PBS + 5% BSA + 1% EDTA) before addition of Brilliant Stain Buffer (BD) and surface staining at 40 °C for 30 min (Supplementary Table 1). Cells were washed and resuspended in Cell Fixation Buffer (eBioscience) at 40 °C overnight. Cells were re-washed and human serum and saponin added to samples 5 min before the addition of cytokine-specific antibodies (Supplementary Table 2) and incubation in the dark at room temperature for 30 min. Cells were washed twice with MACS and run on a BD Symphony A3 flow cytometer (BD Biosciences) with analysis carried out using FlowJo v10.7.1 (Supplementary Fig. 3). Cells that appeared in both the IFN-γ-positive and IL-2-positive gates were taken as the dual-positive IFN-γ^+^IL-2^+^ cells.

### High-throughput live virus microneutralization assay

The B.1.617.2 (Delta) isolate was MS066352H (GISAID accession EPI_ISL_1731019), and was kindly provided by W. Barclay, Imperial College London, through the Genotype-to-Phenotype National Virology Consortium (G2P-UK). The BA.1 (Omicron) isolate was M21021166, and was kindly provided by G. Screaton, University of Oxford, through G2P-UK.

All viral isolates were propagated in Vero V1 cells. Briefly, 50% confluent monolayers of Vero V1 cells were infected with the given SARS-CoV-2 strains at a multiplicity of infection of approximately 0.001. Cells were washed once with DMEM (Sigma, D6429), then 5 ml virus inoculum made up in DMEM was added to each T175 flask and incubated at room temperature for 30 min. DMEM + 1% FCS (Biosera, FB-1001/500) was added to each flask. Cells were incubated at 37 °C, 5% CO_2_ for 4 d until an extensive cytopathogenic effect was observed. Supernatant was harvested and clarified by centrifugation at 2,000 r.p.m. for 10 min in a benchtop centrifuge. Supernatant was aliquoted and frozen at −80 °C. The full protocol for variant virus culture and microneutralization assay is available from Wu et al.^30^.

High-throughput live virus microneutralization assay was run as previously described^31^. Specifically, Vero E6 cells (Institut Pasteur) at 90–100% confluency in 384-well plates (Greiner) were infected with SARS-CoV-2 variants at a multiplicity of infection of <1 in the presence of participants’ serum samples in serial dilutions. Cells were fixed with 4% final formaldehyde, blocked and permeabilized with 3% BSA + 0.2% Triton X-100 in PBS (vol/vol), and infected cells stained using a Biotin-CR3009 antibody (produced in-house), which specifically detects SARS-CoV-2 N-protein, and Streptavidin–Alexa Fluor 488 (Invitrogen). Cellular DNA was detected using DAPI. An Opera Phenix (Perkin Elmer) automated microscope was used to image whole wells at ×5 and the fluorescent areas calculated using the Phenix-associated Harmony software (Perkin Elmer). The sample IC_50_ against a variant was estimated by fitting a four-parameter dose-response curve using SciPy and reported as the fold-dilution of serum samples required to inhibit 50% of detected infection, with additional annotation if the result lies outside the quantitative range (complete inhibition, no inhibition, weak inhibition).

### Statistical analysis

All data were checked for normality using the Kolmogorov–Smirnov test. For comparative analysis of two groups, a Mann–Whitney test was applied. For paired data, two-tailed paired *t*-tests were applied. For comparative analysis with three or more groups, a Kruskal–Wallis test was used. For multiple comparisons, an uncorrected Dunn’s test was used for non-parametric data. Spearman’s rank correlation coefficients were calculated and tested for correlations. *P* values < 0.05 were considered to be statistically significant.

The cumulative incidence of breakthrough infection following the third vaccination dose based on LFD or PCR testing was estimated and Kaplan–Meier curves were plotted. Participants entered the cohort 7 d following the date of their third vaccination dose and were censored at the date of the final PCR or LFD test if infection did not occur, or on the date of fourth vaccine administration (*n* = 5). Participants who had not undergone testing with PCR or LFD after vaccination were excluded from this analysis (*n* = 36). The cumulative incidence of infection was also compared between those aged 65 years and over and those under 65 using the log-rank test.

In a subset of participants who had undergone blood sampling between 7 and 50 d following the third vaccination dose (taken as time of peak vaccine response), spike-specific antibody and cellular response values were modeled against breakthrough infection using linear regression against time from vaccine dose. Participants were grouped into those with spike antibody responses above and below the predicted median titer and those with cellular responses above and below the predicted median level for their time point of observation. The cumulative risk of infection was compared separately against spike-specific antibody titer or the spike-cellular immunity using Kaplan–Meier curves. Participants entered this cohort analysis at 50 d following the third vaccination dose and exited at the date of a positive LFD/PCR test or were censored at the last recorded test date. Separate Cox proportional hazards models were constructed to estimate the hazard ratios of breakthrough infection based on the level of humoral and cellular immunity against spike antigen (high or low).

Data analysis was performed in Graph Pad Prism v9.1.0 and in Stata v16.0.

### Reporting summary

Further information on research design is available in the Nature Portfolio Reporting Summary linked to this article.

## Supplementary information

Supplementary Information Supplementary Figs. 1–9, Discussion and Tables 1–3.

Reporting Summary

## Figures and Tables

**Extended Data Fig. 1:**
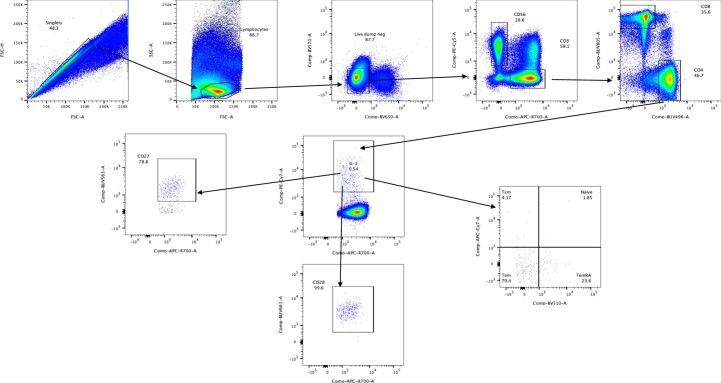
Gating strategy. Gating strategy showing detailing how spike specific CD4 cells were isolated and their memory phenotype as well as CD28/27 status was determined. This was carried out for both IFNy, IL-2 and double positive CD4 cells in response to spike peptide stimulation.

## Data Availability

De-identified test results and limited metadata will be made available for use by researchers in future studies, subject to appropriate research ethical approvals, once the VIVALDI study cohort has been finalized.All source data for this manuscript can be requested from the corresponding author Paul Moss. Additional data are accessible via the Health Data Research UK Gateway (https://www.healthdatagateway.org/).
