# Supplementary material for: Strong peak immunogenicity but rapid antibody waning following third vaccine dose in older residents of care homes
Source: Nat Aging. 2023 Jan 20;3(1):93–104. doi: 10.1038/s43587-022-00328-3 (PMC10154221; doi:10.1038/s43587-022-00328-3)
Supplement: Supplementary file 1 — Supplementary Figs. 1–9, Discussion and Tables 1–3. [file 43587_2022_328_MOESM1_ESM.pdf]

nature aging

Article

https://doi.org/10.1038/s43587-022-00328-3

Strong peak immunogenicity but rapid antibody waning following third vaccine dose in older residents of care homes

In the format provided by the authors and unedited

Supplementary Data

	Staff	Resident	Total	% of total
**Total participants**	220 (103+)	268 (144+)	488	100
**Age >=80**	2 (1+)	207 (108+)	209 (109+)	42.8 (52.2+)
**Age 65-79**	30 (8+)	57 (32+)	87 (40+)	17.8 (46+)
**Age <= 64**	188 (94+)	4 (4+)	192 (98+)	39.3 (51+)
**Median age in years (IQR)**	51 (39-61)	87 (80-92)	73 (55-87)	
**Female**	197 (88+)	184 (100+)	381 (188+)	78.1 (49.3+)
**Male**	23 (15+)	84 (44+)	107 (59+)	21.9 (55.1+)
**BNT162b2 recipients**	178 (83+)	109 (61+)	287 (144)+	58.8 (69.2+)
**ChAdOx1 recipients**	42 (20+)	159 (83+)	201 (103+)	41.2 (51.2+)
**Total LTCFs**	74			
**Mean participants / LTCF (SD)**	6.59 (6.41)			

Supplementary Table 1: Cohort demographics of post 2nd dose recipients
+ Indicates SARS-CoV-2 prior infection
published previously (4)

Antigen	Clone	Fluorophore	Supplier	Code	Amount used per sample (µL)
Viability	N/A	FVS575V	BD	565694	1
CD14	M5E2	BV650	Biolegend	301836	2.5
CD19	HIB19	BV650	Biolegend	302238	1
CD3	SK7	AF700	Biolegend	344822	1
CD8	SK1	BUV805	BD	612889	1
CD4	SK3	BUV496	BD	612936	1
CD27	L128	BUV563	BD	748705	1
CD25	2A3	BUV615	BD	612996	1
CD127	HIL-7R-M21	BUV737	BD	612794	2.5
CD45RA	HI100	BV480	BD	566114	1
CCR7	2-L1-A	APC-Cy7	Biolegend	353212	5
CD69	FN50	BV711	BD	563836	2.5
HLA-DR	G46-6	BV786	BD	564041	2.5
TCRgd	11F2	BB700	BD	745944	2.5
CD56	B159	PE-Cy5	BD	555517	5
CD28	CD28.2	BUV661	BD	741635	2.5

Supplementary table 2: Cell surface staining panel. Red indicates dump channel

Antigen	Clone	Fluorophore	Supplier	Code	Amount used per sample (μL)
IFNγ	B27	BUV395	BD	563563	5
IL-2	MQ1-17H12	PE-Cy7	Biolegend	500326	2
TNF	MAB11	BV750	BD	566359	5
GrzB	GB11	FITC	BD	560211	10
IL-10	JES3-9D7	PE	Biolegend	501404	5
CD107-a	H4A3	BV605	Biolegend	328634	5

Supplementary table 3: Intracellular cytokine staining panel

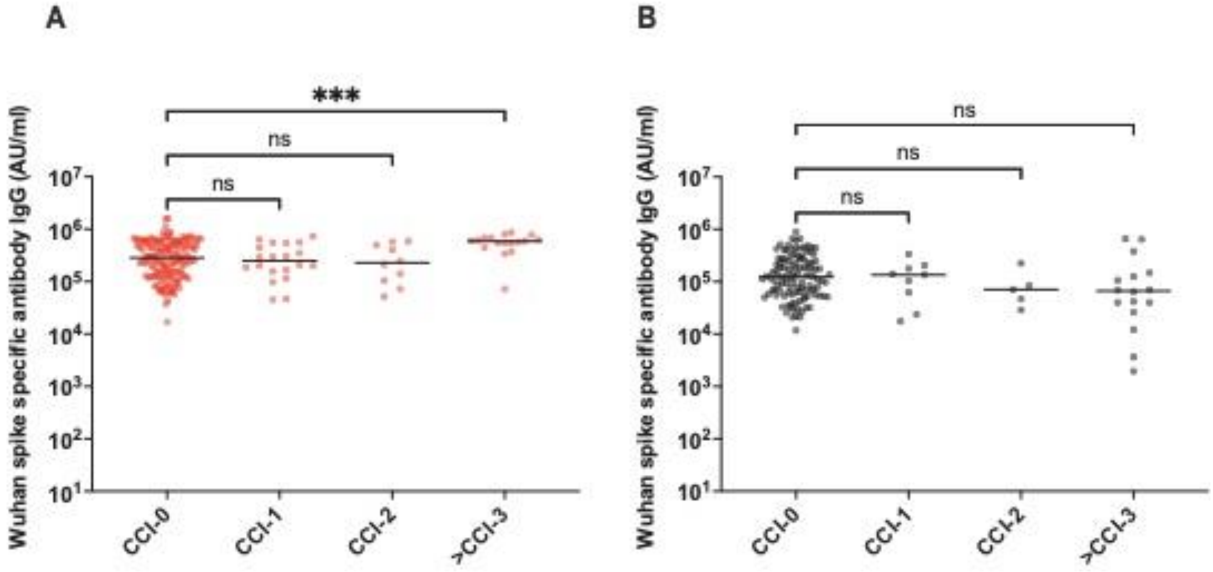

Supplementary Figure 1: Spike-specific antibody response following third booster dose is influenced by Charlson Comorbidity Index (CCI) only in prior infected individuals. A. Prior infected donors Kruskal-Wallis (Uncorrected Dunn's test) *** p=0.0004 n= 198 B. infection-naive donors, Kruskal-Wallis (Uncorrected Dunn's test) n= 143

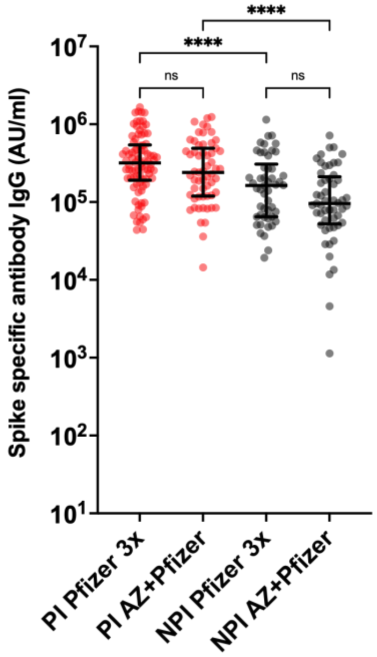

Supplementary Figure 2: Primary series vaccine regimen is not a significant determinant of humoral response following third dose vaccine

A) Wuhan spike-specific antibody titre after 3 COVID-19 vaccine doses in relation to prior infection status and stratified by baseline primary series vaccine regimen. (Pfizer=BNT162b2; AZ=ChAdOx1). Red dots indicate participants with prior natural infection and black dots indicate non-infected donors. Kruskal-Wallis (Uncorrected Dunn's test) ****p=<0.0001 n=248. Black line indicates median antibody titre. Error bards are IQRs

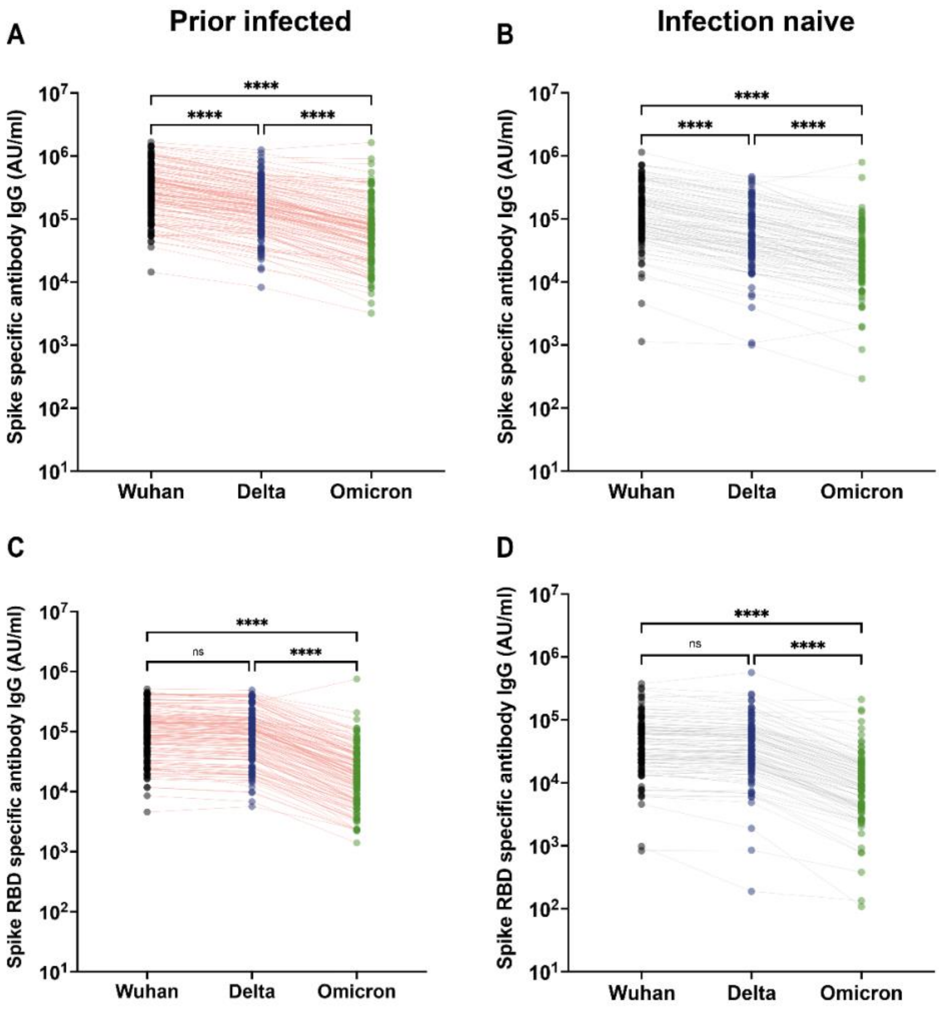

Supplementary Figure 3: Antibody titres to spike protein from Delta and Omicron are lower than those against Wuhan following third vaccine dose.

Post vaccine sera were applied to MSD plates containing whole spike protein or the RBD domain of the Wuhan, Delta or Omicron variants. Lines indicate responses from the same participant.

A) Whole spike-specific binding in prior infected donors. Kruskal-Wallis (Uncorrected Dunn's test) **** p=<0.0001, n=145.

B) Whole spike-specific binding within infection-naive donors. Kruskal-Wallis (Uncorrected Dunn's test) **** p=<0.0001, n=103.

C) RBD-specific binding in prior infected donors. Kruskal-Wallis (Uncorrected Dunn's test) **** p=<0.0001 n.s=not significant n=145.

D) RBD-specific binding within infection-naive donors. Kruskal-Wallis (Uncorrected Dunn's test) **** p=<0.0001 n.s=not significant n=103.

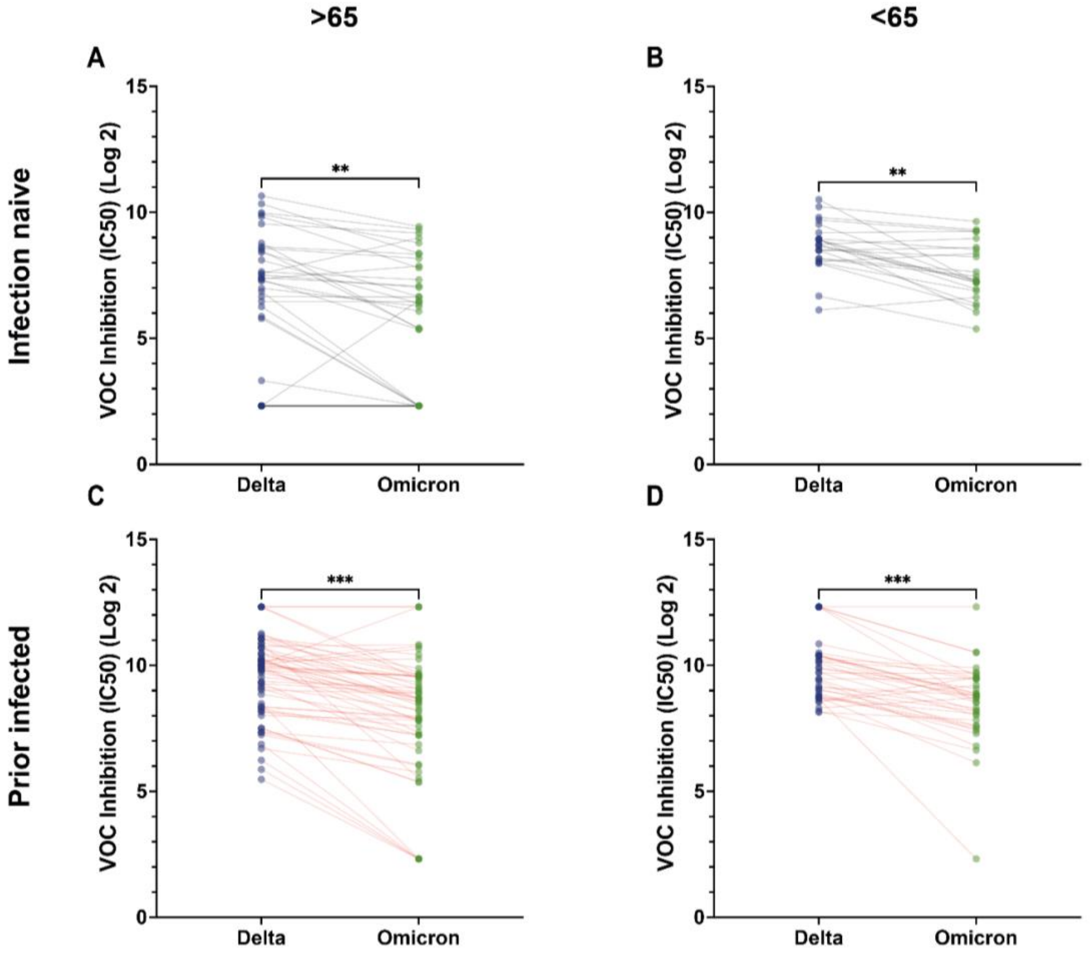

Supplementary Figure 4: Viral neutralisation of Omicron BA.1 is lower than Delta regardless of age or infection status.

A) Paired viral neutralisation titres following a third vaccine in infection-naïve over 65s. Paired t-test, **p=0.0026 n=34

B) Paired viral neutralisation titres following a third vaccine in infection-naïve under 65s. Paired t-test, **p=0.0012 n=23

C) Paired viral neutralisation titres following a third vaccine in prior infected over 65s. Paired t-test, ***p=0.001 n=58

D) Paired viral neutralisation titres following a third vaccine in prior infected under 65s. Paired t-test, ***p=0.0005 n=37

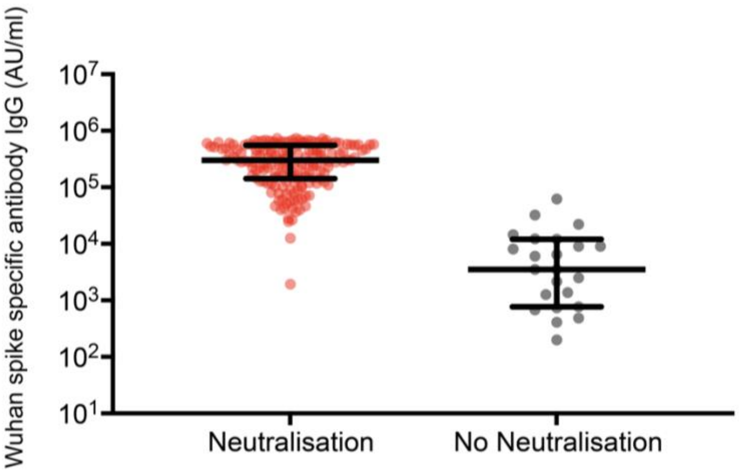

Supplementary Figure 5: Spike specific antibody levels in donors who showed detectable antibody neutralisation of Delta variant compared to those with no neutralisation
Neutralisation data was available on 195 donors of which 22 showed no detectable neutralisation against Delta varint (IC50=5). Median spike-specific antibody response was 3519 AU/ml (IQR: 768 - 12025). Median value in those with neutralisation was 300789 AU/ml (IQR: 142710-550512).

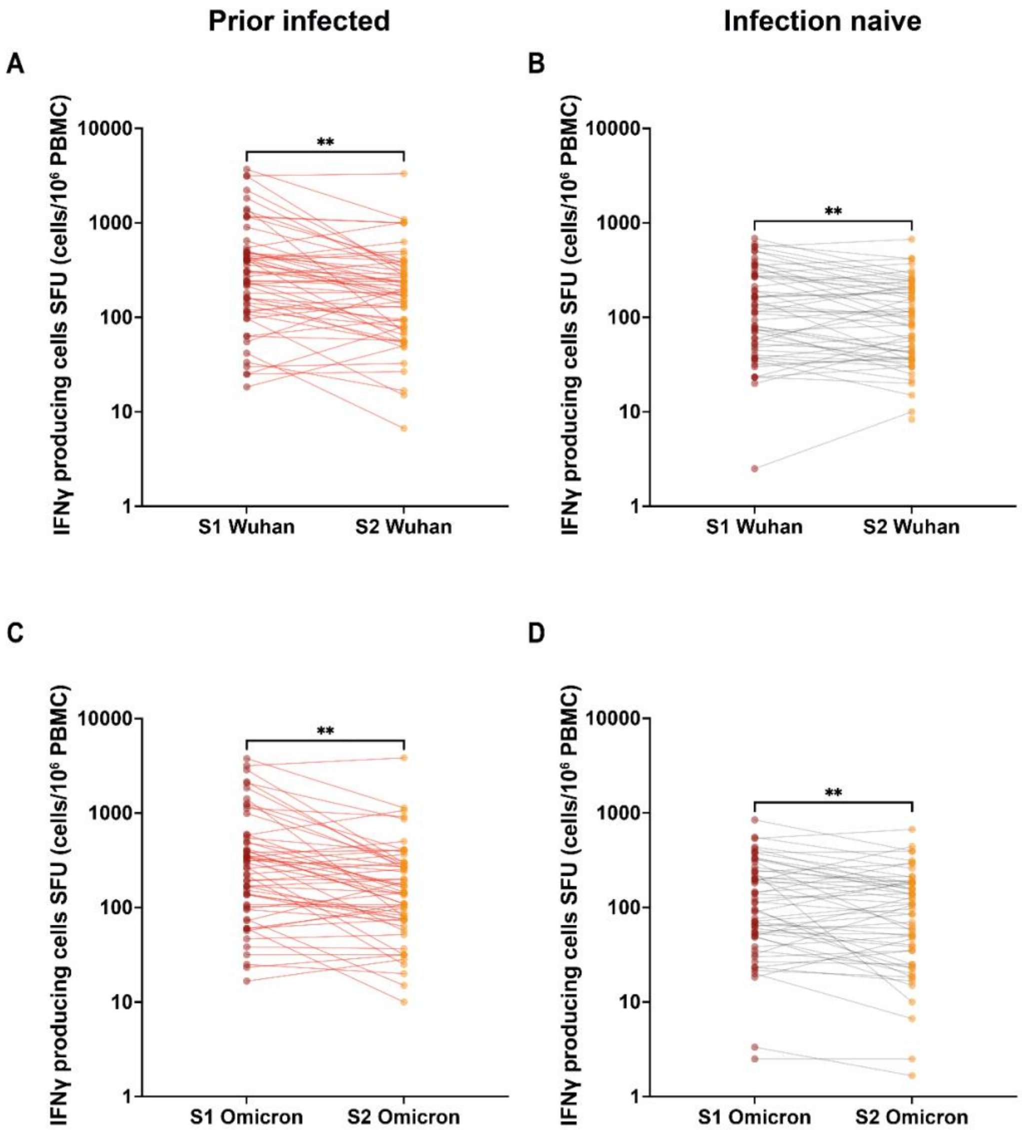

Supplementary Figure 6: IFN-γ ELISpot responses against Wuhan and Omicron S1 peptides are increased compared to Wuhan and Omicron S2 peptides.

A) Paired IFN-γ ELISpot responses to S1 and S2 Wuhan peptides in previously infected donors. Paired t-test, **p=0.0021. n=57

B) Paired IFN-γ ELISpot responses to S1 and S2 Wuhan peptides in infection-naïve donors. Paired t-test, **p=0.0031. n=57

C) Paired IFN-γ ELISpot responses to S1 and S2 Omicron peptides in infection-naïve donors. Paired t-test, **p=0.0016. n=57

D) Paired IFN-γ ELISpot responses to S1 and S2 Omicron peptides in infection-naïve donors. Paired t-test, **p=0.0099. n=57

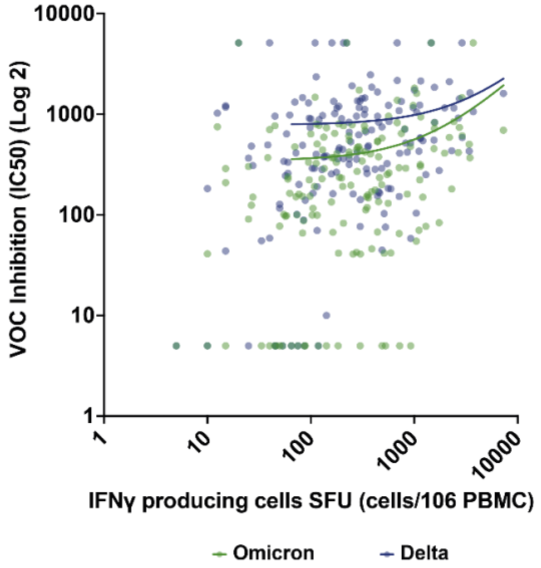

Supplementary Figure 7: Spike-specific cellular response correlates to inhibition of Delta and Omicron variants.
IFN-γ ELISpot spike-specific responses to total Wuhan spike correlated to VOC inhibition. Blue dots and line (Spearman's correlation r=0.32, p=<0.0001, n=162) indicate Delta neutralisation and green dots and line (Spearman's correlation r=0.33, p =<0.0001, n=162) indicate Omicron neutralisation. All fitted lines are linear regressions

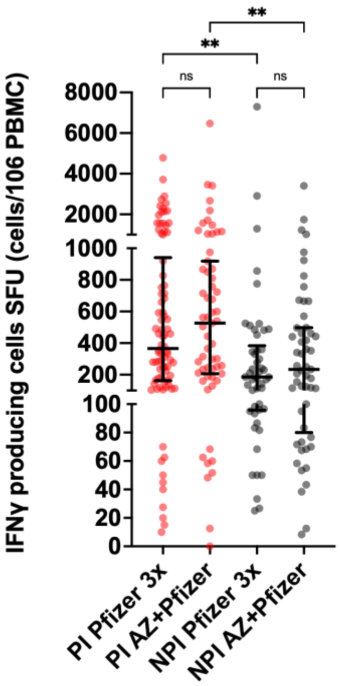

Supplementary Figure 8: Primary series vaccine regimen is not a significant determinant of cellular response following third dose vaccine
Wuhan spike-specific cellular response after 3 COVID-19 vaccine doses in relation to prior infection status and stratified by baseline primary series vaccine regimen. (Pfizer=BNT162b2; AZ=ChAdOx1). Red dots indicate participants with prior natural infection and black dots indicate non-infected donors. Kruskal-Wallis (Uncorrected Dunn’s test) ***p=<0.001 n=248. Black line indicates median antibody titre. Error bards are IQRs

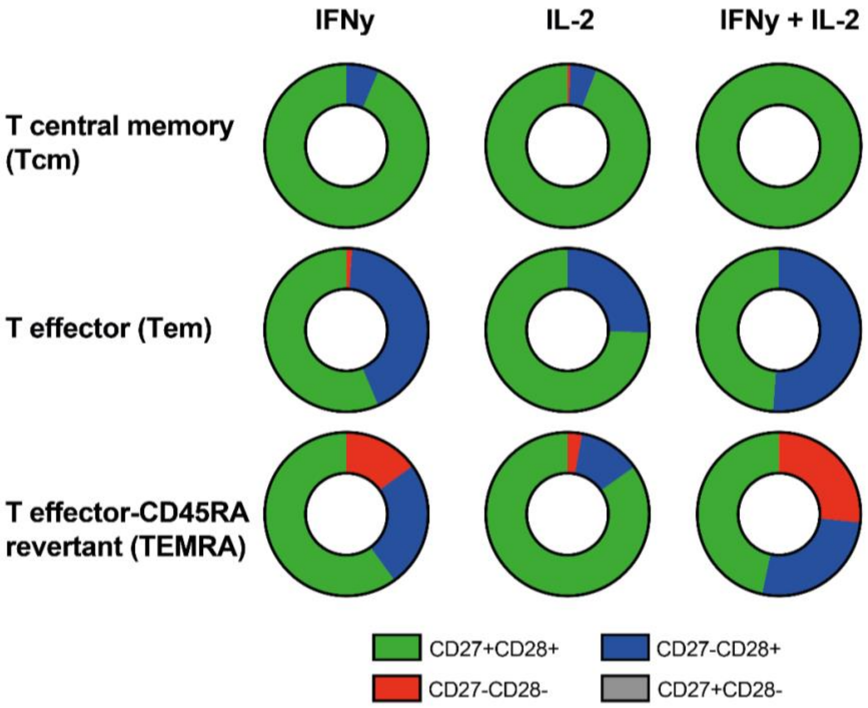

Supplementary figure 9: Distribution of CD27-CD28-, CD27+CD28-, CD27-CD28+ and CD27+CD28+ expressing cells within Central Memory (Tcm), Effector (Tem) and Effector-CD45RA (TemRA) memory pools in relation to single or dual IL-2 and IFN-γ cytokine production by spike-specific CD4+ T cells (n=22). (Tcm IFN-γ: CD27-CD28+ and CD27+CD28+ = 6.5% and 93.5% respectively. Tcm IL-2: CD27-CD28-, CD27+CD28-, CD27-CD28+ and CD27+CD28+ = 0.6%, 0.05%, 5.2% and 94.1% respectively. Tcm IL-2+IFN-γ+: CD27+CD28+ = 100%. Tem IFN-γ: CD27-CD28-, CD27-CD28+ and CD27+CD28+ = 1.1%, 42.5% and 56.3% respectively. Tem IL-2: CD27-CD28+ and CD27+CD28+ = 25.5% and 74.5% respectively. Tem IL-2+IFN-γ+: CD27-CD28+ and CD27+CD28+ = 51.2% and 48.8% respectively. TemRA IFN-γ: CD27-CD28-, CD27-CD28+ and CD27+CD28+ = 15%, 25% and 60% respectively. TemRA IL-2: CD27-CD28-, CD27-CD28+ and CD27+CD28+ = 2.9%, 12.3% and 84.8% respectively. TemRA IL-2+IFN-γ+: CD27-CD28-, CD27-CD28+ and CD27+CD28+ = 26.7%, 26.7% and 46.7% respectively.) Chi-Square p = 0.053 Tcm CD27-CD28+ IFNy, IL-2 and IFN-γ+IL-2+ vs Tcm CD27+CD28+ IFNy, IL-2 and IFN-γ+IL-2+. Chi-Square p = 0.0006 Tem CD27-CD28+ IFNy, IL-2 and IFN-γ+IL-2+ vs Tem CD27+CD28+ IFNy, IL-2 and IFN-γ+IL-2+. Chi-Square p <0.0001 TemRA CD27-CD28- IFNy, IL-2 and IFN-γ+IL-2+ vs TemRA CD27-CD28+ IFNy, IL-2 and IFN-γ+IL-2+ vs CD27+CD28+ IFNy, IL-2 and IFN-γ+IL-2+.
