# Supplementary material for: Strong peak immunogenicity but rapid antibody waning following third vaccine dose in older residents of care homes
Source: Nat Aging. 2023 Jan 20;3(1):93–104. doi: 10.1038/s43587-022-00328-3 (PMC10154221; doi:10.1038/s43587-022-00328-3)
Supplement: Supplementary file 2 — Reporting Summary [file 43587_2022_328_MOESM2_ESM.pdf]

nature portfolio

Corresponding author(s): Dr Gokhan Tut

Last updated by author(s): Dec 9, 2022

Reporting Summary

Nature Portfolio wishes to improve the reproducibility of the work that we publish. This form provides structure for consistency and transparency in reporting. For further information on Nature Portfolio policies, see our Editorial Policies and the Editorial Policy Checklist.

Statistics

For all statistical analyses, confirm that the following items are present in the figure legend, table legend, main text, or Methods section.

n/a	Confirmed	
☐	☒	The exact sample size (*n*) for each experimental group/condition, given as a discrete number and unit of measurement
☐	☒	A statement on whether measurements were taken from distinct samples or whether the same sample was measured repeatedly
☐	☒	The statistical test(s) used AND whether they are one- or two-sided<br>*Only common tests should be described solely by name; describe more complex techniques in the Methods section.*
☒	☐	A description of all covariates tested
☐	☒	A description of any assumptions or corrections, such as tests of normality and adjustment for multiple comparisons
☐	☒	A full description of the statistical parameters including central tendency (e.g. means) or other basic estimates (e.g. regression coefficient) AND variation (e.g. standard deviation) or associated estimates of uncertainty (e.g. confidence intervals)
☐	☒	For null hypothesis testing, the test statistic (e.g. *F*, *t*, *r*) with confidence intervals, effect sizes, degrees of freedom and *P* value noted<br>*Give P values as exact values whenever suitable.*
☒	☐	For Bayesian analysis, information on the choice of priors and Markov chain Monte Carlo settings
☒	☐	For hierarchical and complex designs, identification of the appropriate level for tests and full reporting of outcomes
☐	☒	Estimates of effect sizes (e.g. Cohen's *d*, Pearson's *r*), indicating how they were calculated

Our web collection on statistics for biologists contains articles on many of the points above.

Software and code

Policy information about availability of computer code

Data collection	All data was generated from these software: BD FACS DIVA v9.1 Methodological Minds (MSD), Biosensor 500 (ELISPOT)
Data analysis	All Statistical analysis was done on Graphpad Prism 9 v9.1.0 and FlowJo v10.7.1, SATA v16 and MSD Discovery Workhbench v4.0

For manuscripts utilizing custom algorithms or software that are central to the research but not yet described in published literature, software must be made available to editors and reviewers. We strongly encourage code deposition in a community repository (e.g. GitHub). See the Nature Portfolio guidelines for submitting code & software for further information.

Data

Policy information about availability of data

All manuscripts must include a data availability statement. This statement should provide the following information, where applicable:

- Accession codes, unique identifiers, or web links for publicly available datasets
- A description of any restrictions on data availability
- For clinical datasets or third party data, please ensure that the statement adheres to our policy

Provide your data availability statement here.

Human research participants

Policy information about [studies involving human research participants and Sex and Gender in Research.](#)

Reporting on sex and gender	No sex based analysis was carried out. 267 females and 74 males recruited
Population characteristics	This population was from LTCFs. Total of 341 participants. 183 Staff members and 158 residents. Further breakdown can be found in the manuscript
Recruitment	Eligible LTCFs were identified by the Care Provider's Senior Management Team, or by the National Institute for Health Research (NIHR) Clinical Research Network. Pseudonymised clinical (vaccination status, PCR test results, hospitalisation, death) and demographic (age, sex, staff member versus resident) data were retrieved for staff and residents from participating LTCFs through national surveillance systems. All participants provided written informed consent for blood sample collection or if residents lacked the capacity to consent, a personal or nominated consultee was identified to act on their behalf. No selection bias was possible as participants were recruited across England and all researchers had no access to participant information besides the meta-data used for analysis
Ethics oversight	Blood sampling was carried out from 25th May 2021 until 23rd of February 2022. Ethical approval for this study was obtained from the South Central - Hampshire B Research Ethics Committee, REC Ref: 20/SC/0238.

Note that full information on the approval of the study protocol must also be provided in the manuscript.

Field-specific reporting

Please select the one below that is the best fit for your research. If you are not sure, read the appropriate sections before making your selection.

☒ Life sciences ☐ Behavioural & social sciences ☐ Ecological, evolutionary & environmental sciences

For a reference copy of the document with all sections, see [nature.com/documents/nr-reporting-summary-flat.pdf](#)

Life sciences study design

All studies must disclose on these points even when the disclosure is negative.

Sample size	A total o 341 participants were used in our study and this included 107 over 80 years old, 51 between 65-80 years old and 183 under 65 years old, across 48 different LTCFs in England. The cohort represents the care home populations well in England. Samples were chosen based on the sample collection occurring for the wider VCIVALDI study. No statistical analysis was done for determining sample size. This is one of the largest studies to look at booster vaccine responses in LTCFs
Data exclusions	The VIVALDI study (ISRCTN14447421) is a prospective cohort study which was set up to investigate SARS-CoV-2 transmission, infection outcomes and immunity in residents and staff in LTCFs in England that provide residential and/or nursing care for adults aged 65 years and over (https://wellcomeopenresearch.org/articles/5-232/v2).<br>Eligible LTCFs were identified by the Care Provider's Senior Management Team, or by the National Institute for Health Research (NIHR) Clinical Research Network. Pseudonymised clinical (vaccination status, PCR test results, hospitalisation, death) and demographic (age, sex, staff member versus resident) data were retrieved for staff and residents from participating LTCFs through national surveillance systems. All participants provided written informed consent for blood sample collection or if residents lacked the capacity to consent, a personal or nominated consultee was identified to act on their behalf. No thoer data was excluded besides what is stated above
Replication	All samples were run in duplicates at least and we have more sample left if anyone wanted to retest our samples.
Randomization	All donors were assigned a ID number and only information that was essential for analysis (e.g.age) was included. Participants were goruped based on age and prior infection status
Blinding	All investigators were blinded to the cohort

Reporting for specific materials, systems and methods

We require information from authors about some types of materials, experimental systems and methods used in many studies. Here, indicate whether each material, system or method listed is relevant to your study. If you are not sure if a list item applies to your research, read the appropriate section before selecting a response.

Materials & experimental systems

n/a	Involved in the study
☐	☒ Antibodies
☐	☒ Eukaryotic cell lines
☒	☐ Palaeontology and archaeology
☒	☐ Animals and other organisms
☒	☐ Clinical data
☒	☐ Dual use research of concern

Methods

n/a	Involved in the study
☒	☐ ChIP-seq
☐	☒ Flow cytometry
☒	☐ MRI-based neuroimaging

Antibodies

Antibodies used

Antigen	Clone	Fluorophore	Supplier	Code	Amount used per sample (μL)
Viability	N/A	FVS575V	BD	565694	1
CD14	M5E2	BV650	Biolegend	301836	2.5
CD19	HIB19	BV650	Biolegend	302238	1
CD3	SK7	AF700	Biolegend	344822	1
CD8	SK1	BUV805	BD	612889	1
CD4	SK3	BUV496	BD	612936	1
CD27	L128	BUV563	BD	748705	1
CD25	2A3	BUV615	BD	612996	1
CD127	HIL-7R-M21	BUV737	BD	612794	2.5
CD45RA	HI100	BV480	BD	566114	1
CCR7	2-L1-A	APC-Cy7	Biolegend	353212	5
CD69	FN50	BV711	BD	563836	2.5
HLA-DR	G46-6	BV786	BD	564041	2.5
TCRgd	11F2	BB700	BD	745944	2.5
CD56	B159	PE-Cy5	BD	555517	5
CD28	CD28.2	BUV661	BD	741635	2.5
IFNγ	B27	BUV395	BD	563563	5
IL-2	MQ1-17H12	PE-Cy7	Biolegend	500326	2
TNF	MAB11	BV750	BD	566359	5
GrzB	GB11	FITC	BD	560211	10
IL-10	JES3-9D7	PE	Biolegend	501404	5
CD107-a	H4A3	BV605	Biolegend	328634	5

SULFO-Tag anti-human IgG antibody -supplied from MSD with the MSD kit, used 1:1000

Validation

All antibodies validated by the supplier. All antibodies were tested and titrated using healthy donor PBMC. Data not shown

Eukaryotic cell lines

Policy information about cell lines and Sex and Gender in Research

Cell line source(s)

VERO E6 from Dr. Bjorn Meyer, Institut Pasteur, Paris, France.

Authentication

Not authenticated.

Mycoplasma contamination

Tested negative for Mycoplama contamination.

Commonly misidentified lines
(See ICLAC register)

N/A

Flow Cytometry

Plots

Confirm that:

☒ The axis labels state the marker and fluorochrome used (e.g. CD4-FITC).

☒ The axis scales are clearly visible. Include numbers along axes only for bottom left plot of group (a 'group' is an analysis of identical markers).

☒ All plots are contour plots with outliers or pseudocolor plots.

☒ A numerical value for number of cells or percentage (with statistics) is provided.

Methodology

Sample preparation	1.5x10^6 PBMC were stimulated with either SARS-CoV-2 Spike S1 and S2 peptide pool at a final concentration of 2ng/ml per peptide for 6 hours. Protein transport inhibitor and CD107a-specific antibody were added after 1 hour and PBMC washed with MACS (PBS+5% BSA+1% EDTA) prior to addition of Brilliant Stain Buffer (BD) and surface staining at 4oC for 30 minutes (supplementary table 1). Cells were washed and resuspended in Cell Fixation Buffer (eBioscience) at 4oC overnight. Cells were re-washed and human serum and saponin added to samples 5 minutes before the addition of cytokine-specific antibodies (supplementary table 2) and incubation in the dark at room temperature for 30 minutes. Cells were washed twice with MACS and run on a BD Symphony A3 flow cytometer (BD Biosciences) with analysis carried out using FlowJo v10.7.1 software (supplementary figure 3) (FlowJo). Cells that appeared in both the IFN-γ and IL-2 positive gates were taken as the dual positive IFN-γ+IL-2+ cells.
Instrument	BS Symphony A3 Flow cytometer (BD Biosciences)
Software	FlowJo v 10.7.1
Cell population abundance	No cell sorting was done
Gating strategy	Singlets were chosen first (FSC-H vs FSC), then lymphocytes (SSC vs FSC), then live cells while removing monocytes (Live vs dump), then CD3 positive cells were isolated to get CD4 and CD8 cells. Gating strategy available in supplementary

☒ Tick this box to confirm that a figure exemplifying the gating strategy is provided in the Supplementary Information.
